# Feasibility and Diagnostic Yield of Endoscopic Ultrasonography-Guided Fine Needle Biopsy With a New Core Biopsy Needle Device in Patients With Gastric Subepithelial Tumors

**DOI:** 10.1097/MD.0000000000001622

**Published:** 2015-10-09

**Authors:** Minju Lee, Byung-Hoon Min, Hyuk Lee, Sangjeong Ahn, Jun Haeng Lee, Poong-Lyul Rhee, Jae J. Kim, Tae Sung Sohn, Sung Kim, Kyoung-Mee Kim

**Affiliations:** From the Department of Pathology and Translational Genomics (ML, SA, K-MK); Department of Medicine (B-HM, HL, JHL, P-LR, JJK); and Department of Surgery, Samsung Medical Center, Sungkyunkwan University School of Medicine, Seoul, Korea (TSS, SK).

## Abstract

As treatment decisions for patients with gastric subepithelial tumors (SETs) largely depend on the histopathologic diagnosis, noninvasive and effective tissue acquisition methods are definitely required for proper management of gastric SETs. Recently, a new endoscopic ultrasonography-guided fine needle biopsy (EUS-FNB) device with ProCore reverse bevel technology was developed. We aimed to elucidate the feasibility and diagnostic yield of EUS-FNB with this new core biopsy needle device in patients with gastric SETs.

A prospectively maintained database was retrospectively reviewed to identify consecutive patients who underwent EUS-FNB with a 22-gauge ProCore needle for gastric SETs 2 cm or larger. The main outcome measurement was the diagnostic yield of EUS-FNB. Procedure results were categorized into diagnostic, suggestive, or nondiagnostic.

Of the 43 patients, needle punctures were successful in all cases irrespective of tumor location. EUS-FNB procedure results were diagnostic in 86.0%, suggestive in 4.7%, and nondiagnostic in 9.3% of cases, respectively. The diagnostic yield was the highest in fundus (100.0%), followed by body (89.5%), cardia (83.3%), and antrum (50.0%). All 18 patients with cardiac SET were finally diagnosed to have leiomyoma, and 16 patients with diagnostic or suggestive results avoided surgery. A heterogeneous echo pattern on EUS was found in 33.3% of cases with nondiagnostic or suggestive results and in 5.4% with diagnostic results. In multivariate analysis, no independent predictor of unsuccessful EUS-FNB with nondiagnostic or suggestive results was identified. Agreement between EUS-FNB and surgical pathology was 100% with respect to the diagnosis of gastrointestinal stromal tumor. However, there was a significant discrepancy in mitotic counts observed between the EUS-FNB and surgical specimens in patients with gastrointestinal stromal tumor. There were no significant procedure-related adverse events during and after the procedures.

EUS-FNB with a 22G ProCore needle is a technically feasible, safe, and effective procedure for pathologic diagnosis of gastric SETs. This procedure can help refine surgical indications and facilitate a proper treatment decisions for gastric SETs, especially in the cardia.

## INTRODUCTION

The prevalence of gastric subepithelial tumors (SETs) detected during routine upper endoscopy ranges from 0.3% to 1.0%.^1,2^ Gastric SETs include a diverse array of benign, potentially malignant, and malignant lesions.^3–6^ Gastrointestinal stromal tumors (GISTs) are the most common potentially malignant or malignant gastric SETs and account for 70 to 75% of gastric hypoechoic SETs larger than 2 cm.^7^ Lymphoma, carcinoid tumors, and even metastatic carcinoma can present as gastric SETs. Surgical resection is usually recommended for gastric GIST larger than 2 cm, and other malignant lesions require specific therapy according to pathologic diagnosis,^8,9^ making accurate differential diagnosis of gastric SET crucial for proper management.

Endoscopic ultrasonography-guided fine needle aspiration (EUS-FNA) and trucut biopsy (EUS-TCB) are 2 representative noninvasive methods of tissue acquisition from gastric SETs. EUS-FNA allows harvesting of representative material for cytopathological evaluation from most gastric SETs. However, the amount of cytological material obtained by EUS-FNA is often insufficient for the immunohistochemical staining required to differentiate GISTs from other benign gastric mesenchymal tumors.^9^ Because of this limitation, the diagnostic yield of EUS-FNA for gastric SET is limited, ranging from 34% to 79%.^10–13^ EUS-TCB has emerged as a method to address the limitations of EUS-FNA. EUS-TCB provides larger-core tissue specimens that preserve tissue architecture and allow histological examination with immunohistochemistry. However, EUS-TCB is associated with technical difficulties because of the use of a stiff 19-gauge (G) needle. The diagnostic yield of EUS-TCB for gastric SET is not superior to EUS-FNA, ranging from 47% to 63%.^2,11,12^ On the basis of these data, recent European guidelines recommend EUS-FNA or EUS-TCB for patients with gastric SETs only in limited indications.^9^

Recently, a new EUS-guided fine needle biopsy (EUS-FNB) device with ProCore reverse bevel technology was developed. This new core biopsy needle device was designed to obtain cytological aspirates and histological core samples simultaneously, and has shown promising results for pancreatic tumors or lymph nodes.^14–19^ However, data are still limited on the feasibility and efficacy of EUS-FNB with this new needle device in patients with gastric SETs.^16,20^

In the present study, we aimed to elucidate the feasibility and diagnostic yield of EUS-FNB with a new core biopsy needle device in patients with gastric SETs 2 cm or larger in size.

## PATIENTS AND METHODS

### Patients

We retrospectively reviewed a prospectively maintained database to identify all consecutive patients who underwent EUS-FNB for gastric SETs 2 cm or larger in size from July 2013 to April 2015. During the study period, all EUS-guided tissue acquisitions from gastric SET were done with EUS-FNB procedures. In our institution, EUS-FNB is not indicated when gastric SET is diagnosed as a vascular or cystic lesion or lipoma based on EUS findings. All enrolled patients provided written informed consent before the EUS-FNB procedure. The study protocol was approved by the institutional review board at Samsung Medical Center.

### Outcome Measurements

The main outcome measurement was the diagnostic yield of EUS-FNB. Procedure results were categorized as follows: diagnostic, if biopsy specimens or cytological aspirates were deemed adequate by the pathologist for making a diagnosis, including immunohistochemical staining whenever necessary; suggestive, if sufficient samples were obtained for cytology, and a suggestive primary diagnosis was assigned, but a definitive final diagnosis was not achieved; and nondiagnostic, if samples were primarily insufficient for diagnosis.^21^

### Study Procedures

Two experienced endoscopists (B.-H.M. and H.L.) performed all EUS-FNB with a conventional linear array Echoendoscope (GF-UCT260, Olympus Medical Systems, Tokyo, Japan). All punctures were done with a 22G ProCore needle (EchoTip ProCore; Cook Medical, Limerick, Ireland) under the guidance of real-time EUS imaging. After the needle passed into the lesion, the endosonographer moved the needle back and forth in the lesion 25 to 30 times while an assistant simultaneously pulled out the stylet slowly and continuously over 40 to 50 seconds. The simultaneously withdrawn stylet generated minimal negative pressure; thus, a suction syringe was not applied during the procedure. Biopsy specimens were expressed onto glass slides by flushing air into the needle assembly. The needle passes were repeated until enough biopsy specimens (visible cores) had been obtained, as determined by gross inspection of the endosonographer. Biopsy specimens were placed into a formalin bottle. Cytologic smears were also done with aspirated specimens by the endosonographer. In our institution, an on-site cytopathologist is not available during the EUS-FNB procedure. Smeared slide glasses were fixed in an absolute alcohol solution. Biopsy and aspiration slides of all cases were diagnosed by an experienced gastrointestinal pathologist (K.-M.K.) and reviewed by 2 independent pathologists (M.J.L. and S.J.A.) for the study. Immunohistochemical staining was performed for differential diagnosis of gastric SET whenever necessary. For spindle cell lesion in H&E slides, immunohistochemical study for c-kit, desmin, and S-100 protein was performed in all cases to confirm pathologic diagnosis of GIST, leiomyoma, and schwannoma, as previously described.^22^ For GIST, mitotic figures were counted in all fields of the biopsy specimen, and this mitotic count was compared with mitotic counts on wedge resection specimens in cases of surgery.

### Statistical Analysis

Categorical data were analyzed using the χ^2^ test or Fisher exact test. Continuous data were analyzed using the Student *t* test or Mann–Whitney *U* test. Univariate and multivariate logistic regression analysis was performed to identify the independent predictors of unsuccessful EUS-FNA with nondiagnostic or suggestive result. All statistical analyses were performed using Statistical Package for Social Science (SPSS Inc, Chicago, IL). *P* values less than 0.05 were considered statistically significant.

## RESULTS

### Baseline Characteristics of Gastric SETs and Procedures

From July 2013 to April 2015, 43 patients underwent EUS-FNB for gastric SETs 2 cm or larger in size. Table 1 summarizes patient demographic and baseline characteristics of gastric SETs and EUS-FNB procedures. The most common location of gastric SETs was the body, followed by the cardia. The majority of tumors showed a homogeneous hypoechoic echo pattern and seemed to originate from the muscularis propria layer on EUS.

**TABLE 1:**
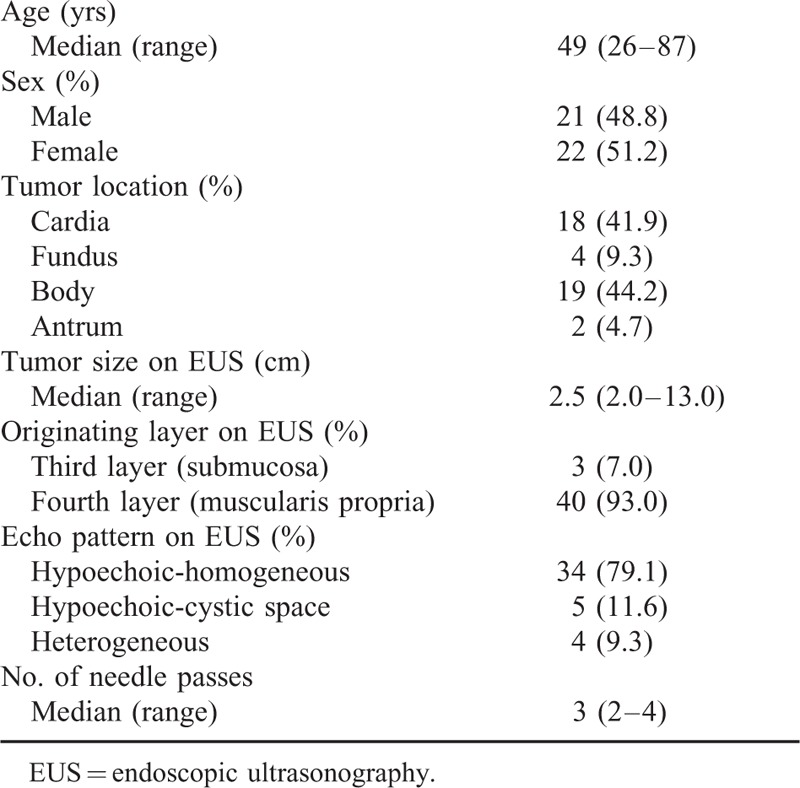
Patient Demographic and Characteristics of Gastric Subepithelial Tumors and Procedures

**FIGURE 1:**
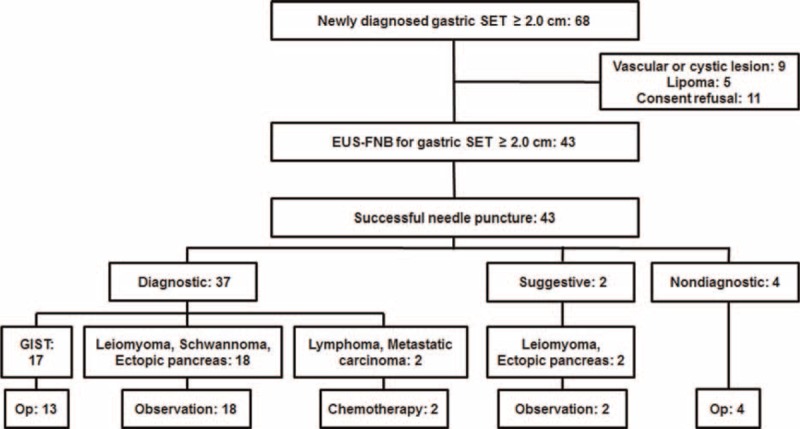
Flow chart of 43 patients with a gastric subepithelial tumor undergoing endoscopic ultrasonography-guided fine needle biopsy with a 22G ProCore needle. EUS-FNB, endoscopic ultrasonography-guided fine needle biopsy; GIST, gastrointestinal stromal tumor; Op, operation; SET, subepithelial tumor.

Endoscopic ultrasonography-guided fine needle biopsy procedures with a 22G ProCore needle were technically feasible and needle punctures were successful in all cases irrespective of tumor location (Figure 1). The median number of needle passes was 3 (range 2–4). There were no significant procedure-related adverse events during and after procedures.

### Diagnostic Yield

**FIGURE 2:**
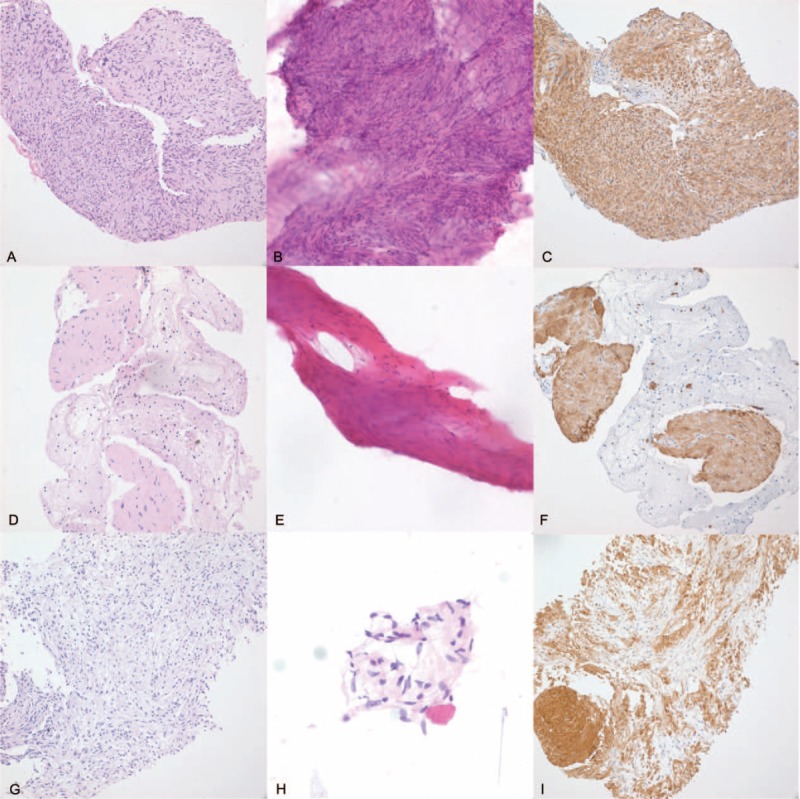
Representative histologic findings of endoscopic ultrasonography-guided fine needle biopsy specimens of gastric subepithelial tumors. Biopsy (A) and aspiration (B) specimens of gastrointestinal stromal tumor are composed of characteristic spindle cells with high cellularity and perinuclear vacuoles (200×, H&E). The tumor cells are positive for c-kit (C). Leiomyoma in biopsy (D) and aspiration (E) specimens shows spindle cells with low cellularity, extracellular collagen globules, and lacks significant cytologic or architectural atypia (200×, H&E). The tumor cells are positive for desmin (F). Schwannoma with low-to-moderate cellularity with lymphoid cuff in biopsy (G) and aspiration (H) specimens (200×, H&E). The tumor cells are positive for S100 protein (I).

Endoscopic ultrasonography-guided fine needle biopsy procedure results were diagnostic in 86.0% (37/43), suggestive in 4.7% (2/43), and nondiagnostic in 9.3% (4/43) of the cases, respectively (Figure 1 and Table 2**)**. The most common pathologic diagnosis was GIST followed by leiomyoma. Typical pathologic features of gastric SETs are shown in Figure 2. All 4 patients with nondiagnostic results had hypoechoic tumors originating from the muscularis propria layer on EUS and underwent wedge resection as GIST could not be ruled out. Final pathologic diagnoses based on surgical specimens were leiomyoma in 2 cases, Schwannoma in 1 case, and ectopic pancreas in 1 case. Among 37 SETs with diagnostic results, 94.5% of cases could be diagnosed with core biopsy samples alone (Table 3).

**TABLE 2:**
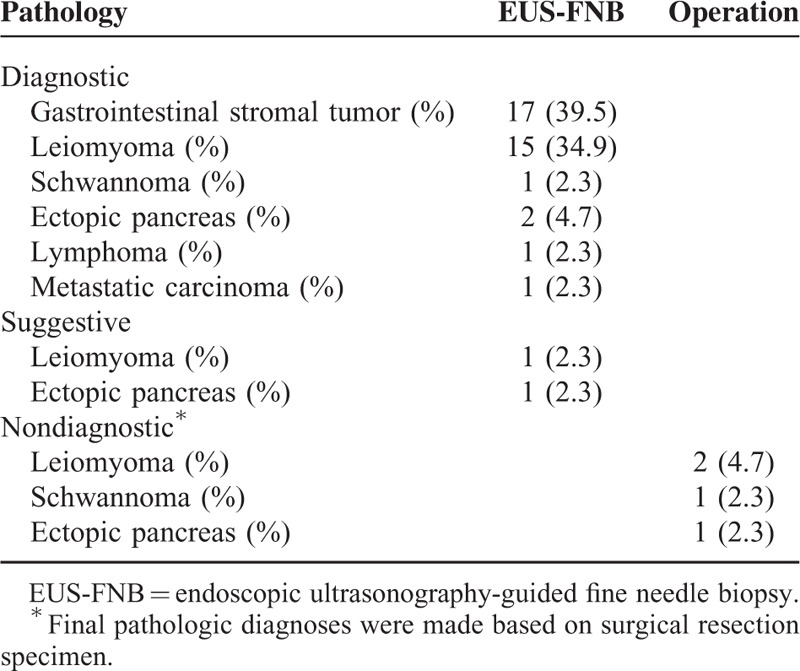
Pathologic Results of Gastric Subepithelial Tumor Diagnosed by EUS-Guided Fine Needle Biopsy or Surgery

**TABLE 3:**
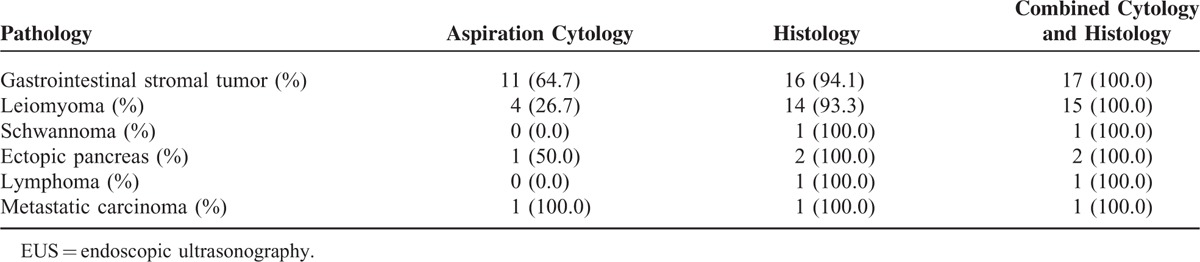
Contribution of Aspiration Cytology and Histology to the Diagnosis Made by EUS-guided find needle biopsy

### Factors Affecting Diagnostic Yield

Table 4 shows the diagnostic yield of the EUS-FNB procedure according to tumor location. The diagnostic yield was the highest in the fundus (100.0%), followed by the body (89.5%), cardia (83.3%), and antrum (50.0%). All SETs in the cardia were diagnosed as leiomyoma. There were 2 SET cases in the antrum. One case was diagnosed as metastatic carcinoma by EUS-FNB. The other case underwent surgery, as the EUS-FNB result was nondiagnostic. This case was finally diagnosed as ectopic pancreas based on the surgical specimen. The sizes of SETs on EUS were 4.9 cm for metastatic carcinoma and 2.9 cm for ectopic pancreas, respectively. Number of needle pass was 3 in both cases.

**TABLE 4:**
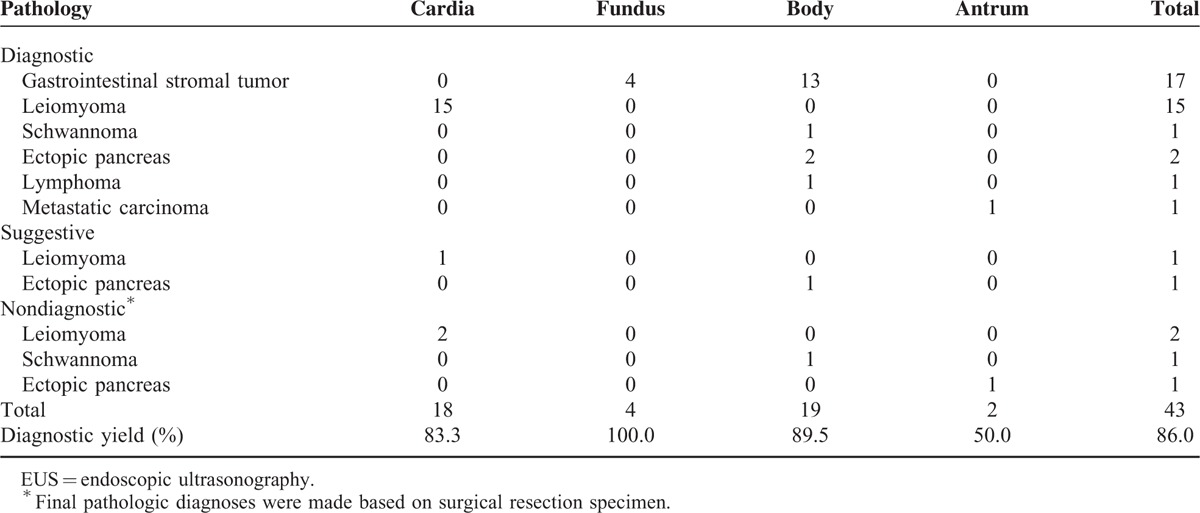
Diagnostic Yield of EUS-guided Fine Needle Biopsy According to Tumor Location

Cases with diagnostic results did not display significant differences in tumor location, tumor size on EUS, or originating layer on EUS compared to cases with nondiagnostic or suggestive results. A heterogeneous echo pattern on EUS was found in 33.3% of cases with nondiagnostic or suggestive results and 5.4% of cases with diagnostic results, although this difference did not reach statistical significance (Table 5). In the multivariate analysis, no independent predictor of unsuccessful EUS-FNB with nondiagnostic or suggestive results was identified.

**TABLE 5:**
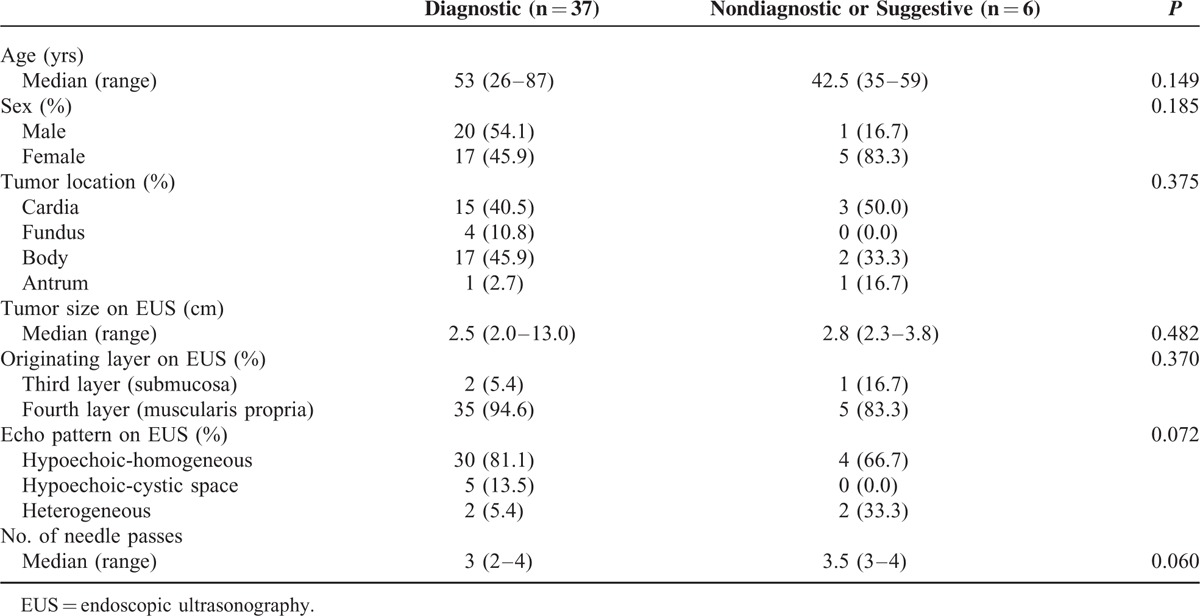
Comparison of Features of Gastric Subepithelial Tumors With and Without Diagnostic Pathologic Results Through EUS-guided Fine Needle Biopsy

### Correlation Between EUS-FNB and Surgical Pathology in Patients With GIST

Among 17 patients diagnosed as having GIST by EUS-FNB, 2 patients received imatinib, 2 patients were referred to other hospitals, and 13 patients underwent wedge resection, including 1 patient who received neoadjuvant imatinib therapy before surgery (Figure 1). The final surgical pathology was GIST in all 13 patients, consistent with the results based on their EUS-FNB specimens.

Among the 13 patients undergoing wedge resection for GIST, mitotic counts of the core biopsy specimen obtained through EUS-FNB were evaluated in 12 patients, except 1 patient, whose diagnosis of GIST was made based on aspiration cytology. The number of observed high-power fields (HPFs) for mitotic count examination ranged from 1 to 11, which was far smaller than the required number of 50. No mitotic figures were seen in core biopsy specimen from any of these 12 patients. However, in their corresponding surgical specimen, we found mitoses in 11 patients, ranging from 1 to 13 per 50 consecutive HPFs. Only 1 patient did not show mitotic figures in surgical specimen. Among the remaining 4 cases not undergoing surgery, mitoses were observed in core biopsy specimens from 2 patients with large tumors (2 mitotic counts in 14 HPFs in a 9.0-cm–sized GIST; 1 mitotic count in 12 HPFs in a 10.0-cm–sized GIST).

## DISCUSSION

As treatment decisions for patients with gastric SETs largely depend on the histopathologic diagnosis, noninvasive and effective tissue acquisition methods are definitely required for proper management of gastric SETs. However, EUS-FNA and EUS-TCB procedures provide only limited diagnostic yield for gastric SETs.^7,9^ In contrast to EUS-FNA and EUS-TCB, the present study showed that EUS-FNB with a 22G ProCore needle was technically feasible and highly effective for core tissue acquisition from gastric SETs 2 cm or larger in size. Needle punctures were successful in all cases irrespective of tumor location and the diagnostic yield was up to 86.0%. If including suggestive results, EUS-FNB with a 22G ProCore needle could guide treatment decisions in 90.7% of cases. No significant procedure-related adverse events occurred during and after the procedures.

To date, only a few studies have assessed the efficacy and safety of EUS-FNB with a ProCore needle for gastric SETs. The study by Kim et al^20^ included 12 patients undergoing EUS-FNB with a 22G ProCore needle for esophageal, gastric, and duodenal SETs. In their study, diagnostic yield of EUS-FNB for upper gastrointestinal SETs was 75%. Iglesias-Garcia et al^16^ evaluated the efficacy of 19G ProCore needle in 11 patients with upper gastrointestinal SETs and achieved correct diagnosis in 81.8% of SET cases. Both of these studies were limited in that they included small heterogeneous study population with esophageal and duodenal SETs, as well as gastric SETs. In contrast, the present study focused on gastric SETs, which enabled a detailed analysis of the feasibility and diagnostic yield of EUS-FNB with a 22G ProCore needle for gastric SETs.

In the present study, the diagnostic yield was over 80% in the fundus, body, and cardia. However, diagnostic yield for SET in the antrum was only 50%. This study included only 2 cases of antral SET, and therefore this figure in the antrum was not conclusive and may underestimate the true diagnostic capability. As needle punctures were successful in all cases included in the study, the low diagnostic yield in the antrum was likely associated with a feature of gastric SET itself, such as low cellularity rather than tumor location. In our study, the nondiagnostic case in the antrum was finally diagnosed as ectopic pancreas. Ectopic pancreas is characterized by benign pancreatic duct, acini, or islet cells, and has lower cellularity compared to typical gastric SET like GIST,^5^ which might prevent harvesting of sufficient core tissue during the EUS-FNB procedure. We also found that cases with nondiagnostic or suggestive results were more frequently associated with a heterogeneous echo pattern on EUS than cases with diagnostic results (33.3% vs 5.4%), although the difference did not reach statistical significance. In the present study, all 4 cases with heterogeneous echo patterns were diagnosed as ectopic pancreas (2 cases by diagnostic result, 1 case by suggestive result, and 1 case based on surgical specimen).

The present study included the 18 cases of SET in the cardia. Interestingly, all 18 patients with cardiac SET were diagnosed to have leiomyoma (15 cases by diagnostic result, 1 case by suggestive result, and two cases based on surgical specimen) and 16 patients with diagnostic or suggestive results avoided surgery. This finding of dominance of leiomyoma in the cardia was consistent with results from recent studies.^3,23^ Our group reported that leiomyomas are the most common tumor type in the cardia, accounting for 63.6% of cardia SETs.^3^ Lee et al^23^ also reported that leiomyomas account for 55.2% of cardiac SETs, whereas GISTs account for 41.4%. Given this high prevalence of leiomyoma in the cardia, and the technical difficulty and potential complications associated with surgery, EUS-FNB should be positively considered for gastric SET in the cardia to avoid unnecessary surgery.

In this study, we counted mitosis in both EUS-FNB and corresponding surgical resection specimens in 12 patients undergoing wedge resection for GIST. The surgical resection specimens showed variable mitotic counts. However, there were no mitotic figures on the corresponding EUS-FNB specimens regardless of tumor size or risk of malignant behavior. Polkowski et al^2^ and Ricci et al^24^ reported similar discrepant results. They found no correlation of mitotic count between EUS-FNA or TCB specimens and surgical specimens. Our results suggest that mitotic count cannot be reliably evaluated with EUS-FNB with a 22G ProCore needle despite the high diagnostic yield of the method overall. The discrepancy in mitotic count evaluation may be caused by the limited amount of tissue obtained from EUS-guided tissue acquisition.

This study was limited in that it was performed at a single tertiary referral center and had a retrospective design. An on-site cytopathologist was not available during EUS-FNB procedures. However, most diagnostic results could be made with the core biopsy specimen alone. In addition, a recently suggested algorithm did not recommend the routine use of on-site cytopathology evaluation for EUS-FNB.^25^ The major strengths of our study compared to the previous ones^16,20^ were its larger and homogeneous study population, including only patients with gastric SETs, and circumstantial pathologic examination. These features enabled a detailed analysis of the feasibility and diagnostic yield of EUS-FNB with a 22G ProCore needle for gastric SETs.

In conclusion, EUS-FNB with a 22G ProCore needle is a technically feasible, safe, and effective procedure for pathologic diagnosis of gastric SETs 2 cm or larger in size, with an overall diagnostic yield of 86.0%. With this high diagnostic yield, EUS-FNB with a 22G ProCore needle can help refine surgical indications and facilitate a proper treatment decisions for gastric SETs, especially in the cardia.
